# Endodontic variables in patients with SARS-CoV-2 infection (COVID-19) in relation to the severity of the disease

**DOI:** 10.4317/medoral.25773

**Published:** 2023-01-15

**Authors:** Manuel Poyato-Borrego, María León-López, Jenifer Martín-González, José M Cisneros-Herreros, Daniel Cabanillas-Balsera, Juan J Segura-Egea

**Affiliations:** 1 Infectious diseases department. Clinical Unit of infectious diseases, microbiology and parasitology. Hospital universitario virgen del Rocío. Sevilla, Spain; 2 Department of Stomatology, Section of Endodontics, School of Dentistry, University of Sevilla, Sevilla, Spain

## Abstract

**Background:**

Severe acute respiratory syndrome coronavirus 2 (SARS-Cov-2) is the cause of the ongoing coronavirus disease 2019 (COVID-19) pandemic. It has been hypothesized oral health may be related to the severity and complications of COVID-19. The aim of this study was to analyze the prevalence of apical periodontitis and the frequency of root canal treatment in a sample of patients with SARS-CoV-2 infection (COVID-19), correlating them with the severity of the disease.

**Material and Methods:**

This retrospective study was conducted following the Strengthening Reporting Observational Studies in Epidemiology (STROBE) guidelines. The study examined 280 patients with positive real time PCR COVID-19 test whose treatment was performed in our hospital. Fifty-two patients aged 52.3 ± 17.3 years, including 30 males and 22 females, who had an orthopantomography in their clinical record, performed in the last 2 years, were included. Patients with SARS-CoV-2 infection were grouped as mild or moderate (MM) and severe or critical (SC) illness groups, according to the NIH COVID-19 Treatment Guidelines (Wu & McGoogan 2020). Radiographic records were analyzed and apical periodontitis (AP) was diagnosed as radiolucent periapical lesions (RPLs), using the periapical index score (PAI). Student’s t test, χ2 test and multivariate logistic regression were used in the statistical analysis.

**Results:**

The number of carious teeth was significantly higher in the SC group (3.4 ± 4.1), which showed more than twice as many teeth with carious lesions than the MM group (1.4 ± 1.8) (*p* = 0.02). Multivariate regression analysis showed association between the number of carious teeth and the severity of SARS-CoV-2 disease (OR = 1.5; 95% CI = 1.1–2.1; *p* = 0.017). Endodontic status (OR = 7.12; 95% CI = 1.2-40.9; *p* = 0.027) also correlated with the disease severity.

**Conclusions:**

The results suggest that the oral health status of COVID-19 patients correlated with the severity of the SARS-CoV-2 virus infection. Significant association has been found between the severity of COVID-19 disease and the presence of a greater number of teeth with caries lesions, as well as with endodontic status.

** Key words:**Apical periodontitis, caries, COVID-19, root canal treatment, SARS-CoV-2

## Introduction

Severe acute respiratory syndrome coronavirus 2 (SARS-Cov-2) is the cause of the ongoing coronavirus disease 2019 (COVID-19) pandemic (1). Patients with COVID-19 emerge symptoms at 5-6 days after infection, developing mild symptoms in the initial stage for 2 weeks on average but has the potential to develop into severe illness, including a systemic inflammatory response syndrome, acute respiratory distress syndrome (ARDS), multi-organ involvement and shock (2). Factors including age, comorbidities, immune response, radiographic findings, laboratory markers, and indicators of organ dysfunction may individually or collectively predict worse outcomes (3). Chronic lung disease, moderate to severe asthma, severe obesity, diabetes, hypertension, obesity, chronic kidney disease, and liver disease are also at high risk for severe COVID-19 symptoms (4,5).

On the other hand, poor oral health, including periodontal disease (6,7) and apical periodontitis (8-10), is associated to higher prevalence and worsening of systemic diseases such as diabetes, hypertension, coronary heart disease, obesity, and chronic liver disease. Therefore, it has been hypothesized that oral health may be related to the severity and complications of COVID-19 (4). Moreover, it has been reported positive correlation between the radiological dental damage stage and the severity of COVID-19 disease (11,12). However, no study has investigated the association between periapical and endodontic status and the severity of COVID-19.

The aim of this study was to analyze the prevalence of apical periodontitis and the frequency of root canal treatment in a sample of patients with SARS-CoV-2 infection (COVID-19), correlating them with the severity of the disease.

## Material and Methods

This cross-sectional study was conducted following the Strengthening Reporting Observational Studies in Epidemiology (STROBE) guidelines. The Ethics Committee of the University of Sevilla (Spain) approved the protocol (1071-N-21). Each subject signed a consent form after being informed of the nature of the study.

- Patients’ selection

Participants were recruited among patients with COVID-19, with positive real time PCR COVID-19 test, receiving treatment at the Virgen del Rocío University Hospital (Sevilla, Spain) between March 2020 and April 2021. The study examined 280 patients with positive real time PCR COVID-19 test whose treatment for COVID-19 was performed in our hospital. Fifty-two patients aged 52.3 ± 17.3 years, including 30 males and 22 females, who had an orthopantomography in their clinical record, performed in the last 2 years, were included. Patients without ortopantomography were excluded from the study.

- Clinical spectrum and severity illness classification

Patients with SARS-CoV-2 infection were grouped into the following severity of illness categories, according to the NIH COVID-19 Treatment Guidelines (https://www.covid19treatmentguidelines.nih.gov/overview/clinical-spectrum/) (2), as follows:

1) Mild illness: Individuals who have any of the various signs and symptoms of COVID-19 (e.g., fever, cough, sore throat, malaise, headache, muscle pain, nausea, vomiting, diarrhea, loss of taste and smell) but who do not have shortness of breath, dyspnea, or abnormal chest imaging.

2) Moderate illness: Individuals who show evidence of lower respiratory disease during clinical assessment or imaging and who have an oxygen saturation (SpO2 ≥ 94%) on room air at sea level.

3)Severe illness: Individuals who have SpO2 < 94% on room air at sea level, a ratio of arterial partial pressure of oxygen to fraction of inspired oxygen (PaO2/FiO2 < 300 mm Hg), a respiratory rate > 30 breaths/min, or lung infiltrates > 50%.

4) Critical illness: Individuals who have respiratory failure, septic shock, and/or multiple organ dysfunction.

For analytical purposes, disease severity was dichotomized by classifying patients into two groups: the first comprised patients with mild or moderate illness (MM) and the second grouped patients with severe or critical illness (SC).

As relevant systemic diseases that could influence the severity of the SARS-CoV-2 infection and that have been shown to be associated with endodontic variables, the data of each patient regarding diabetes, cardiovascular diseases and smoking habits were recorded.

- Radiographic examination

The radiographic periapical status was diagnosed based on the examination of digital panoramic radiographs of the jaws. Two trained radiographic technicians, with over ten years of experience, took the panoramic radiographs using a digital ortho-pantomograph machine (Promax®, Planmeca, class 1, type B, 80 KHz, Planmeca, Helsinki, Finland).

- Radiographic evaluation

Periapical status was assessed using the “Periapical Index” (PAI) score (13), as described previously (14,15). A score greater than two (PAI ≥ 3) was considered to be a sign of periapical pathology. The worst score of all roots was taken to represent the PAI score for multi-rooted teeth. Teeth were classified as root-filled teeth if they had been filled with radiopaque material in the root canal(s).

The following information was recorded in structured form for each subject: number of teeth present; number of carious teeth, number of restored/filled teeth, number of teeth that have identifiable radiolucent periapical lesions (AP), and number of root-filled teeth (RFT). For analytical purposes, both AP and RFT variables were dichotomized as absent or ≥ 1 AP or RFT.

The presence of periodontal disease was evaluated radiographically assessing alveolar bone loss. The height of the alveolar bone crest was measured from a fixed reference point (the cemento-enamel junction) proximal to all available teeth. Subjects with alveolar bone loss ≥ 4 mm were considered periodontal patients (16).

- Observers’ calibration

Three blinded observers with extensive clinical experience in endodontics examined the radiographs. Before evaluation, the observers participated in a calibration course for the PAI system, which consisted of 100 radiographic images of teeth, some root-filled and some not, kindly provided by Dr. Ørstavik. Each tooth was assigned to 1 of the PAI scores by using visual references (also provided by Dr. Ørstavik) for the 5 categories within the scale (13). After scoring the teeth, the results were compared to a “gold standard atlas”, and a Cohen Kappa was calculated.

Intra-observer reproducibility was evaluated for each examiner. Every observer scored the panoramic radiographs of 10 patients (5 of each group, randomly selected). Then, one month after this first examination, the observer was re-calibrated in the PAI system and repeated the scoring of the radiographs of the same 10 patients. The intra-observer agreement test on PAI scores on the 10 patients produced a Cohen’s Kappa ranging 0.78 - 0.91.

The Cohen’s Kappa for inter-observer variability ranged 0.75 - 0.87. The consensus radiographic standard was the simultaneous interpretation by the three examiners of the panoramic radiograph of each patient (17,18).

- Statistical analysis

The minimal sample size (n = 28) was calculated using the sample size calculator software of the National Center for Advancing Translational Sciences (NIH, UK) (http://www.sample-size.net/sample-size-proportions/) for the comparison of proportions in two independent samples, with continuity correction. They were considered a two-sided significance level of 5% (α = 0.05, Zα = 1.960), and 80% power (β = 0.20, Zβ = 0.842). The estimated prevalence of AP was 40% (19,20). The prevalence of AP and the frequency of RCT were evaluated on the total number of individuals and the total number of teeth. Raw data was entered into Excel (Microsoft Corporation, Redmond, WA). All analyzes were performed in an SPSS environment (Version 11; SPSS, Inc, Chicago, IL). Student's t test, 2 test, and multivariate logistic regression analysis were used to determine the significance of differences between groups. Data are reported as mean ± standard deviation. According to the established significance level, a *p-value* ≤ 0.05 was considered statistically significant.

## Results

Table 1 shows the distribution of the variables analysed in both severity groups of COVIC-19 patients, MM and SC groups. The mean age of the SC (59.3 ± 13.2 yo) was significantly older than that of the MM (47.2 ± 18.6 yo) (*p* = 0.012). There were no significant differences in the gender distribution between both groups (*p* > 0.05). The mean number of teeth of the patients in the SC group (21.8 ± 5.7) was significantly less than that of the patients in the MM group (25.9 ± 5.7) (*p* = 0.013).

Neither diabetes, nor cardiovascular diseases, nor smoking habits showed significant differences between the two groups (*p* > 0.05). In the MM group, 30.0% of the subjects had periodontal disease, while in the SC group 59.1% did (*p* < 0.05).

Regarding the endodontic variables, in the MM group the number of teeth with AP was 1.6 ± 1.9, while in the SCG group it was 1.7 ± 2.1 (*p* > 0.05). The number of RFT was 0.8 ± 2.0 in the MM group and 1.5 ± 2.6 in the SC one (*p* > 0.05). Taking the patient as a reference, the number of subjects with one or more teeth with AP in the MM group was 19 (63.3%) while in the SC group they were 17 (77.3%) (OR = 1.97; 95% CI = 0.57-6.82; *p* > 0.05). At least one RFT was found in nine patients (30.0%) in the MM group, while in the SC group 12 patients (54.5%) showed RFT (OR = 2.80; 95% CI = 0.89 - 8.81; *p* = 0.07).

No significant differences were found in the number of filled teeth (*p* > 0.05). On the contrary, the number of carious teeth was significantly higher in the SC group (3.4 ± 4.1), which showed more than twice as many teeth with carious lesions than the MM group (1.4 ± 1.8) (*p* = 0.02).

To analyse more deeply the possible association between endodontic variables and the severity of Covid-19 disease, multivariate logistic regressions were run. In the multivariate analysis, including age, gender, number of teeth, number of carious teeth, number of filled teeth, diabetes, cardiovascular diseases, smoking habits, periodontal status, endodontic status, and periapical status as independent variables, and taking as dependent variable the severity of SARS-CoV-2 disease (Table 2). Only the number of carious teeth was shown to be associated with the severity of SARS-CoV-2 disease (OR = 1.55; 95% CI = 1.08 - 2.23; *p* = 0.017).

However, to avoid the model becoming too stressed, in the final regression model only six explanatory variables were included: age, a possible confounding variable, and those showing higher OR and lower *p value*, such as number of carious teeth, diabetic status, periodontal status, smoking habits and endodontic status (Table 3). Carious teeth maintained its significant correlation with the severity of SARS-CoV-2 (OR = 1.50; 95% CI = 1.07-2.10; *p* = 0.017), and endodontic status (OR = 7.12; 95% CI = 1.24-40.90; *p* = 0.029) also correlated with the disease severity.

**Table 1:**
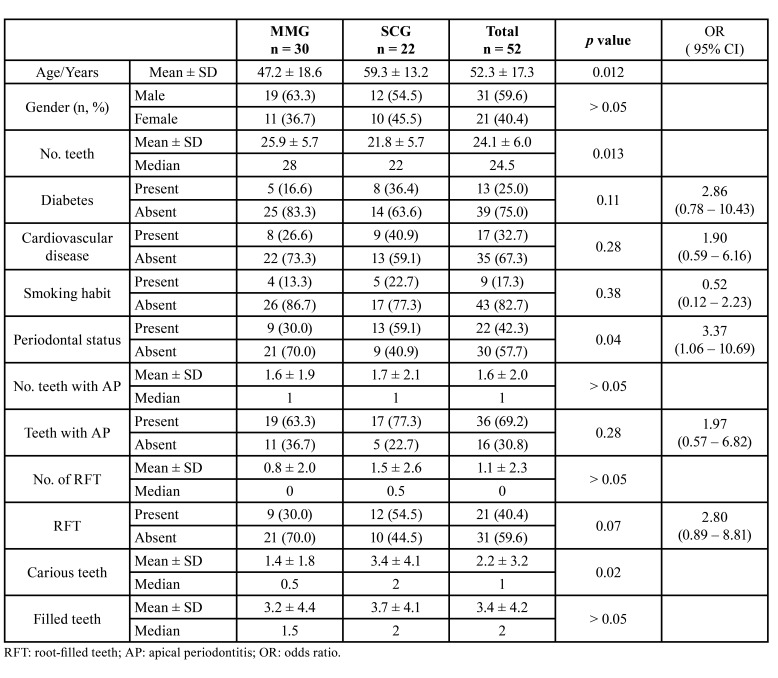
Distribution of analyzed variables among patients with mild / moderate disease (MMG) and severe / critical disease (SCG). Odds ratio (OR) values, and their 95% confidence interval (CI), have been estimated using 2 test.

**Table 2:**
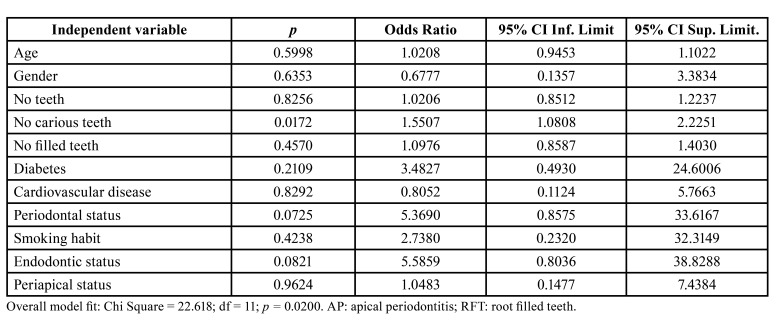
Multivariate logistic regression analyses of the influence of the independent variables age, gender (0 = female; 1 = male), number of teeth, number of carious teeth, number of filled teeth, diabetic status (0 = absent; 1 = present), cardiovascular disease (0 = absent; 1 = present), periodontal status (0 = absent; 1 = present), smoking habits (0 = absent; 1 = present), endodontic status (0 = no RFT; 1 = at least 1 RFT), and periapical status (0 = no tooth with AP; 1 = at least 1 tooth with AP) on the dependent variable “severity of SARS-CoV-2 disease” (0 = mild or moderate; 1 = severe or critical).

**Table 3:**
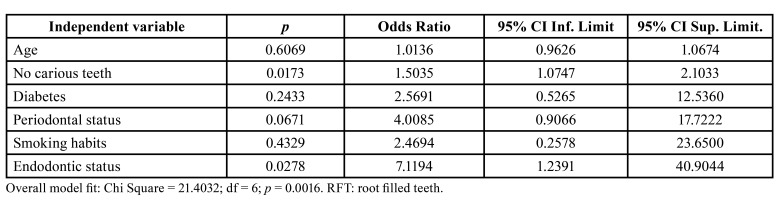
Multivariate logistic regression analyses of the influence of the independent variables number of carious teeth, diabetic status (0 = absent; 1 = present), periodontal status (0 = absent; 1 = present), smoking habits (0 = absent; 1 = present), and endodontic status (0 = no RFT; 1 = at least 1 RFT) on the dependent variable “severity of SARS-CoV-2 disease” (0 = mild or moderate; 1 = severe or critical).

## Discussion

The results of the present study show that the oral health status of COVID-19 patients could be related to the severity of the SARS-CoV-2 virus infection. Although periapical status is not associated with greater severity of the disease, significant association has been found between the severity of COVID-19 and the presence of a greater number of teeth with caries lesions, periodontal disease and endodontic status.

Results show that patients with more severe disease were 8 times more likely to have RFT teeth (OR = 7.12; *p* = 0.029). It can be assumed that patients with a greater number of RFT have a greater history of previous exposure to caries and apical periodontitis. In short, assessing the results of this study as a whole, they suggest the existence of an association between poorer oral health and greater severity of the disease caused by SARS-CoV-2.

Since the study sample had a high risk of COVID-19 transmission, the study was conducted using panoramic radiographs, without including intraoral examination of the patients. To assess the periapical status, the ‘periapical index’ (PAI) scoring system have been used (13), and index widely used in epidemiological and clinical studies (21). Panoramic radiography has the advantages over periapical radiographs of showing all teeth, reducing the patient's exposure to ionizing radiation, faster acquisition, and convenience (22). Although panoramic radiographs have shown good diagnostic accuracy and high specificity in assessing AP, the use of panoramic radiographs has been correlated with an underestimation of the number of periapical lesions (23).

The results of the present study are consistent with those of Sirin & Ozcelik (2021), who also found a correlation between a greater number of caries lesions and greater severity of patients with COVID-19. Likewise, the association between periodontal status and the severity of COVID-19 disease showed in the present study is in agreement with other previous results investigating the possible association between periodontal disease and SARS-Cov-2 infection, concluding that periodontal disease was significantly associated with higher risks of COVID-19 complications (4). Moreover, a case-control study has shown association between periodontal disease and higher risk of ICU admission, need for assisted ventilation and death of COVID-19 patients (24).

The pandemic of SARS-CoV-2 virus has caused a large number of deaths worldwide (25). The risk of death from the disease depends on the immune system, a healthy lifestyle, and physiological and psychological health (26). Taking into account that oral health status is related to systemic health status (10), oral health status could also be related to SARS-Cov-2 infection. On one side, cytokines and microbial antigens released during periodontal or endodontic infection contribute to the systemic pro-inflammatory state, and may influence the development of systemic diseases such as diabetes or cardiovascular diseases (9). Similarly, the pro-inflammatory state caused by oral infections could also contribute to the imbalanced host response to SARS-CoV-2, in which the pro-inflammatory state plays a primary role (27). Another possible link between oral health status and SARS-Cov-2 infection is the presence in the oral mucosa, tongue, and gum epithelium, of ACE2, which is the cellular entry receptor for SARSCoV-2 (28). Poor oral health has been linked to increased expression of ACE2 receptors, leading to the suggestion that good oral hygiene may be important in the Fight against the COVID-19 pandemic (28). Moreover, SARS-CoV-2 has been detected in periodontal pockets and caries lesions, and these sites may act as reservoirs for the virus (29). Patients with a higher number of caries lesions would have a higher viral load and could suffer from more severe disease. Finally, a link between altered dental plaque and oral biofilm on the risk of increased severity of SARS-CoV-2 has been proposed, existing data showing that reducing dental plaque accumulation may minimize COVID-19 severity (30).

The results of this study should be viewed with caution given that this investigation has some limitations. No direct oral examination of the patients has been carried out, but only a radiographic assessment. In addition, there is control group, although this drawback is partially corrected by the multivariate analysis.

The main problem that arises in the interpretation of the results comes from the significant difference in the mean age of the patients in both severity groups, very logical in a disease that is characterized by more severely affecting older patients. Considering that the mean age of the SC (59.3 yo) was significantly older than that of the MM (47.2 yo) (*p* = 0.012), the association observed between the severity of Covid-19 and the state of oral health could also be attributed to the older age of the SC group. However, in the multivariate logistic regression analysis, including age as a co-variable, the association between both variables continued to be significant.

## Conclusions

The present results suggest that the oral health status of COVID-19 patients correlated with the severity of the SARS-CoV-2 virus infection. Significant association has been found between the severity of COVID-19 disease and the presence of a greater number of teeth with caries lesions, as well as with periodontal disease and endodontic status.

The results of this study, along with others already mentioned, should be translated to clinical practice. Thus, evaluation of the oral health status of patients with COVID-19 could help predict the possibility of complications and the level of severity that the disease may have. The information provided by an orthopantomography on the state of oral health could justify its indication at the time of diagnosis of COVID-19, but new studies are required to confirm the prognostic usefulness of orthopantomography in the management of COVID-19 patients.
